# Ensemble machine learning identifies genetic loci associated with future worsening of disability in people with multiple sclerosis

**DOI:** 10.1038/s41598-022-23685-w

**Published:** 2022-11-11

**Authors:** Valery Fuh-Ngwa, Yuan Zhou, Phillip E. Melton, Ingrid van der Mei, Jac C. Charlesworth, Xin Lin, Amin Zarghami, Simon A. Broadley, Anne-Louise Ponsonby, Steve Simpson-Yap, Jeannette Lechner-Scott, Bruce V. Taylor

**Affiliations:** 1 Menzies Institute for Medical Research, University of Tasmania, 17 Liverpool St, Hobart, TAS 7000 Australia; 2 Menzies Health Institute Queensland and School of Medicine, Griffith University Gold Coast, G40 Griffith Health Centre, QLD 4222, Australia; 3 Developing Brain Division, The Florey Institute for Neuroscience and Mental Health, Royal Children’s Hospital, University of Melbourne Murdoch Children’s Research Institute, Parkville, VIC 3052 Australia; 4 Neuroepidemiology Unit, Melbourne School of Population & Global Health, The University of Melbourne, Melbourne, VIC 3053 Australia; 5 Department of Neurology, Hunter Medical Research Institute, Hunter New England Health, University of Newcastle, Callaghan, NSW 2310 Australia

**Keywords:** Genetic association study, Epidemiology, Multiple sclerosis, Machine learning

## Abstract

Limited studies have been conducted to identify and validate multiple sclerosis (MS) genetic loci associated with disability progression. We aimed to identify MS genetic loci associated with worsening of disability over time, and to develop and validate ensemble genetic learning model(s) to identify people with MS (PwMS) at risk of future worsening. We examined associations of 208 previously established MS genetic loci with the risk of worsening of disability; we learned ensemble genetic decision rules and validated the predictions in an external dataset. We found 7 genetic loci (*rs7731626*: *HR* 0.92, *P* = 2.4 × 10^–5^; *rs12211604*: *HR* 1.16, *P* = 3.2 × 10^–7^; *rs55858457*: *HR* 0.93, *P* = 3.7 × 10^–7^; *rs10271373*: *HR* 0.90, *P* = 1.1 × 10^–7^; *rs11256593*: *HR* 1.13, *P* = 5.1 × 10^–57^; *rs12588969*: *HR* = 1.10, *P* = 2.1 × 10^–10^; *rs1465697*: *HR* 1.09, *P* = 1.7 × 10^–128^) associated with risk worsening of disability; most of which were located near or tagged to 13 genomic regions enriched in peptide hormones and steroids biosynthesis pathways by positional and *eQTL* mapping. The derived ensembles produced a set of genetic decision rules that can be translated to provide additional prognostic values to existing clinical predictions, with the additional benefit of incorporating relevant genetic information into clinical decision making for PwMS. The present study extends our knowledge of MS progression genetics and provides the basis of future studies regarding the functional significance of the identified loci.

## Introduction

Multiple sclerosis (MS) is a chronic neurodegenerative disease typified by the accumulation of disability at varying rates^1^. MS occurs in people who have an underlying genetic susceptibility and are exposed to viral and environmental risk factors^2^. While the individual causes of MS are not known, the development of MS involves a complex interplay between genetic and environmental factors, particularly exposure to Epstein-Barr virus (EBV)^3,4^. According to the World Atlas of MS (3rd edition), the number of people living with MS globally has increased from 2.3 million people in 2013 to 2.8 million people in 2020^5,6^. There is currently no cure for MS. The focus has been to develop strategies and interventions to manage or slow disability progression, and to improve the quality of life of affected individuals. Disease modifying therapies (DMTs)^7–10^ and vitamin D treatments (VitD)^11–15^ are currently the only avenues used to prevent relapses, new brain and spinal cord lesions, and perhaps prevent worsening of neurological disability^16,17^.

Significant progress has been made towards elucidating the role of clinical and environmental factors that affects MS disability progression. Particularly, older age, male sex, higher body mass index (BMI), higher number of previous relapses, exposure to higher latitudes, lower median income, higher depression scores, smoking status, higher baseline MRI T2 lesion load (T2L), cerebrospinal fluid (CSF) biomarkers, and neurofilament light chains (NFL), have been shown to predict the MS disease time-course to some degree^18–25^. However, despite these advances, the disease course remains largely unpredictable^26^, with considerable inter- and intra-individual variability^27–30^.

There have been notable proponents for no effect of currently known risk variants on MS outcomes after onset^24,31–36^. There is, however, a plausible effect of genetic variants on MS progression, in particular relating to the severity of primary inflammation and/or relapses^37,38^. Nevertheless, the genetic determinants of disability progression in MS remain elusive. Although the International MS Genetic Consortium (10.1126/science.aav7188?url_ver=Z39.88-2003&rfr_id=ori:rid:crossref.org&rfr_dat=cr_pub%20%200pubmed)^39^ have identified $\sim$ 232 genetic loci to be associated with MS risk, limited studies have been conducted to identify those that predict future worsening of disability^25,40–43^. Additionally, genetic decision rules that can be translated to aid existing clinical and environmental prognostic models in identifying MS subjects prone to future worsening of disability is not yet available.

Notwithstanding, machine learning models have recently been applied in studies of MS disability progression, including standard random forest (RF) and gradient boosting machines (GBM)^21,22,30,36,40,44–50^. Despite their continued use in predicting disability, past and recent studies^15,16^ (not related to MS) have shown that these models have (1) *limited clinical utility* as they rely strictly on a discrete-time evolution of disease processes, meanwhile in MS, disability progression is characterised by a continuous-time evolution of expanded disability status scores (EDSS)^1,9^; (2) *weak predictive power* as they do not account for correlated outcomes^51^ (e.g. correlation due to the sporadic time series of EDSS^21,50^); (3) *lack interpretability* as it is difficult to understand how such models make prediction decisions^52^. Based on lessons learned from precision medicine^53^, RF and GBM are prone to overfitting and selection bias as their internal variable-splitting mechanisms often generates variables with too many possible splits/choices. These models also rely on the property of *normality, independent*, and *identically* distributed outcomes, which are frequently being violated in real-world clinical applications. In addition to RF and GBM classifiers, support vector machines, neural network and deep learning algorithms have similar drawbacks^51,53^.

Recently, Ngufor et al*.*^51^ developed a mixed-effect machine learning (MEML) platform that combined the properties of RF and GBM with generalised mixed-effects regression trees (GMERT) to predict changes in glycaemic control for patients with Type 2 diabetes. Compared to the RF and GBM, their mixed-effect counterparts called MErf and MEgbm, respectively, can learn from complex tree ensembles to produce simple, readable, and interpretable risk models to assist in clinical predictions^47,51^. These models are capable of modeling correlated outcomes (random effects) and linkage disequilibrium (LD) structure between genetic variants (single nucleotide polymorphisms (SNPs)). Compared to standard methods like RF and GBM, MEML models have been shown to have better sensitivities and accuracies in predicting the clinical course of type II diabetes^47,51^.

Using well-established and validated MS genetic loci (risk SNPs) published by IMSGC (10.1126/science.aav7188?url_ver=Z39.88-2003&rfr_id=ori:rid:crossref.org&rfr_dat=cr_pub%20%200pubmed)^39^, we aimed to identify MS genetic loci associated with worsening of disability over time; and to develop and validate simple, learnable and interpretable ensemble genetic learning model(s) and genetic decision rules to identify people with MS (PwMS) at risk of future worsening. To this end, we investigated three hypotheses namely: (1) MS related genetic variants will have additional prognostic values to existing clinical and environmental predictors; (2) Disability worsening based on EDSS scores follows a first-order Markovian process in which future disability is predicated on the prior disability history, and genetic predisposition; (3) MEML models will have better sensitivities in predicting future longitudinal changes in EDSS scores compared to standard RF and GBM.

## Materials and methods

### Data, study cohort, and inclusion criteria

**Figure 1:**
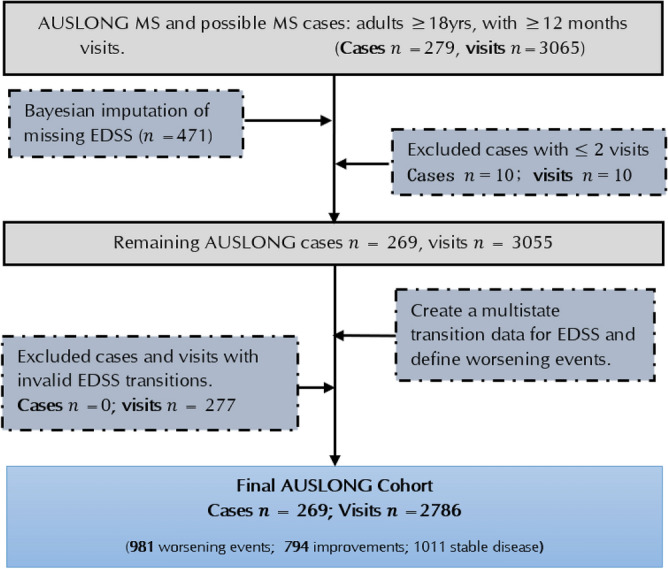
A flow chart of AUSLONG data extraction and case selection criteria.

Using prospective data pooled from the multi-centre (Brisbane, Newcastle, Geelong and Western Victoria, and Tasmania) Australian Longitudinal Cohort Study (the AusLong Study (https://www.menzies.utas.edu.au/research/diseases-and-health-issues/research-projects/the-auslong-study-of-factors-that-contribute-to-the-development-and-progression-of-ms))^54^ of MS, we analysed *279* prospectively assessed first demyelination event (FDE) participants enrolled between 2003 and 2006^55^. The AusLong Study (https://www.menzies.utas.edu.au/research/diseases-and-health-issues/research-projects/the-auslong-study-of-factors-that-contribute-to-the-development-and-progression-of-ms)^54^ has ethical approval from the Tasmanian Health and Medical Research Ethics Committee (ref: H0010499, 01/-5/2009); the Queensland Institute of Medical Research Human Research Ethics Committee (ref: P1252, 22/05/2009); the Royal Brisbane and Women’s Hospital Human Research Ethics Committee (ref: HREC/09/QRBW/299, 19/10/2009); the Hunter New England Human Research Ethics Committee (ref: 09/04/15/5.04, HREC/09/HNE/139, SSA/09/HNE/140, 10/08/2009); and the Barwon Health Human Research Ethics Committee (ref: BH 09/24, BH 03/46, 04/08/2009). All experiments (blood collection, genotyping, and clinical examinations) were conducted in accordance with the guidelines of each committee at the participating centres. Written informed consent was obtained from all subjects and/or their legal guardian(s) in accordance with the Declaration of Helsinki^56^. EDSS scores were acquired prospectively at intervals up to 15 years post FDE by trained and certified neurologists, and a validated telephone EDSS was obtained at yearly computer-assisted telephone interviews from 2 to 3 years post FDE. Initial data extraction (*n* = 279 cases) was done using the revised 2017 McDonalds criteria^57^ in which cases were defined at their last review as either remaining as clinically isolated syndrome (CIS), relapsing-onset MS (ROMS), secondary progressive MS (SPMS), or progressive-onset MS (POMS). The selection criteria for the final cohort were done as illustrated in Fig. 1.

### Genotyping, imputation, and quality control

The Illumina Infinium Global Screening Array-24 v2.0 BeadChip was used to genotype DNA samples from AusLong Study participants. Genotypes were called using Illumina GenomeStudio software. Strict quality control was conducted according to established protocols^58^. In brief, samples were excluded for three reasons: a call rate ≤ 99%, duplicate discordance, or gender error. Further, variants were excluded based on a call rate ≤ 99% or deviation from Hardy–Weinberg equilibrium with *p* < 1.0 × 10^–6^. Two principal components analyses were conducted, one excluding HapMap samples to identify population outliers, and one including HapMap samples to help interpret the outliers^58^. To maximise genetic coverage, the dataset were imputed using the algorithm implemented in IMPUTE version 4^59^ using 1000 Genomes phase 3^60^ as the reference genotype panel (GRCh37/hg19). Genetic variants having an imputation score ≤ 0.5 and minor allele frequency (MAF) ≤ 0.05 were discarded. For the remaining variants, those that were previously identified as being related to MS risk by IMSGC (10.1126/science.aav7188?url_ver=Z39.88-2003&rfr_id=ori:rid:crossref.org&rfr_dat=cr_pub%20%200pubmed)^39^ were extracted (*n* = 208 of 232 SNPs) and considered in the association analysis. To be clearer, this study uses the IMSGC (10.1126/science.aav7188?url_ver=Z39.88-2003&rfr_id=ori:rid:crossref.org&rfr_dat=cr_pub%20%200pubmed)^39^ risk SNP list as a reference source to identify MS risk variants that may also contribute to the risk of worsening of disability.

### Imputation of missing EDSS measures

Imputation of missing EDSS (n = 471 of 3065) was based on a Bayesian approach using the *JointAI* R-package^61^. Conditional on the observed SNPs genotypes, the EDSS scores were considered missing at random. The imputation model is a cumulative logit mixed-effect proportional odds model^1^ defined on 8 disability states (1 = EDSS [0–1.5], 2 = EDSS [2–2.5], 3 = EDSS [3–3.5], 4 = EDSS [4–4.5], 5 = EDSS [5–5.5], 6 = EDSS [6–6.5], 7 = EDSS [7–7.5], and 8 = EDSS [8–9.5]). Based on the results from previous studies^23,25,62–65^, clinical and environmental factors including sex, age at disease onset*,*BMI, titre of Epstein–Barr Nuclear Antigen IgG (EBNA), smoking status, hospital anxiety depression scores (HADS), and previous EDSS scores (EDSSPREV), were used as “analysis variables” to impute EDSS levels, whereas latitude (study site), vitamin D supplementation status (VitD status), and MS disease course subtype (MSTYPE) were used as “auxiliary variables” to inform the imputation of any missing value(s) found in the “analysis variables”. These variables were included in the imputation model following their importance in predicting worsening of disability^25^. In the cumulative logit mixed model, we posit that

1 $$\mathrm{logit}\left(P\left({y}_{ij}>k\right)\right)={\alpha }_{k}+{Z}_{ij}^{T}\beta +{\zeta }_{ij}^{T}{b}_{i}, k\in 1, \dots , K,$$

$${\gamma }_{1}, {\delta }_{1}, \dots , {\delta }_{K-1}\sim N\left({\mu }_{\gamma }, {\sigma }_{\gamma }^{2}\right),$$

$${\mu }_{y}\sim N\left(0, {\sigma }_{u}\right),$$

$${\sigma }_{\gamma }^{2}\sim \Gamma (\varepsilon , \varepsilon )),$$

$${\gamma }_{k}\sim {\gamma }_{k-1}+\mathrm{exp}\left({\delta }_{k-1}\right), k=2,\dots , K,$$

where ${y}_{ij}$ is the EDSS level for subject $i$ at visit $j$, ${\gamma }_{k}$ are 7 intercepts representing the levels of EDSS ($\mathrm{i}.\mathrm{e}., k=2,\dots ,8$), ${Z}_{ij}^{T}$ is a fixed-effect design matrix containing the clinical and environmental covariates including time-varying effects (BMI, HADS, VitD status, and MSTYPE), with a corresponding vector of fixed effects regression coefficients $\beta$; and ${\zeta }_{ij}^{T}$ is a design matrix containing random effects, ${b}_{i}$ are random deviations from the overall intercepts ${\gamma }_{k}$; ${\mu }_{y}$ and ${\sigma }_{\gamma }^{2}$ are hyperpriors^61^.

### Outcome measures, analysis endpoint, and data structure

Based on the study design, a first-order Markov’s assumption for continuous-time EDSS transitions was considered^1,66–68^, and defined as: “the current EDSS state (EDSS score) depends on the previous states (EDSSPREV), and all covariate histories”. In other words, using 8 categories (listed above) of the newly imputed EDSS score, we considered a continuous-time evolution for each disability state, wherein the state at the previous observation is retained until the current visit. Note that an observation may also represent a transition to a different state before arriving at the current state, or a repeated observation of the same underlying state at the end of follow-up.

Using these assumptions, we transformed the data and defined our clinical endpoint to capture continuous-time transitions in disability states as: *y* = 1 denoting “worsening” events (transitions from a lower to a higher disability state) made by an individual from study entry, and *y* = 0 denoting “improved” events (transitions from a higher to a lower disability state). All stable-state transitions or stable disease (no change in EDSS) were excluded as these were considered non-informative censoring events, and could lead to *likelihood drainage,* and potentially alter the results. Therefore, only informative (“improved”) events were censored. The event status for the $i$th subject at the $j$th visit was defined as

$${y}_{ij}=\left\{\begin{array}{l}1,\quad if\, worsening \,events, \\ \\ 0, \quad otherwise \end{array}\right..$$

Since individuals entered the study at different times, we defined the *time-to-worsening* of disability as the time to switch disability states. Specifically, it is the continuous time elapsed since MS diagnosis until the current observation. This was achieved using the “msm2Surv” function in *mstate* R-package^69^. To enable comparison of baseline hazards, the start time for all cases was set to zero at study entry.

### Statistical analysis

All statistical analyses were conducted before (after) imputation of EDSS, respectively. To identify risk SNPs that predicted the *time-to-worsening*, and/or associated with future *worsening* events over time, a three-stage process was employed.

### Stage 1: variable selection, risk estimation, and prognostic modeling

#### Variable selection

**Figure 2:**
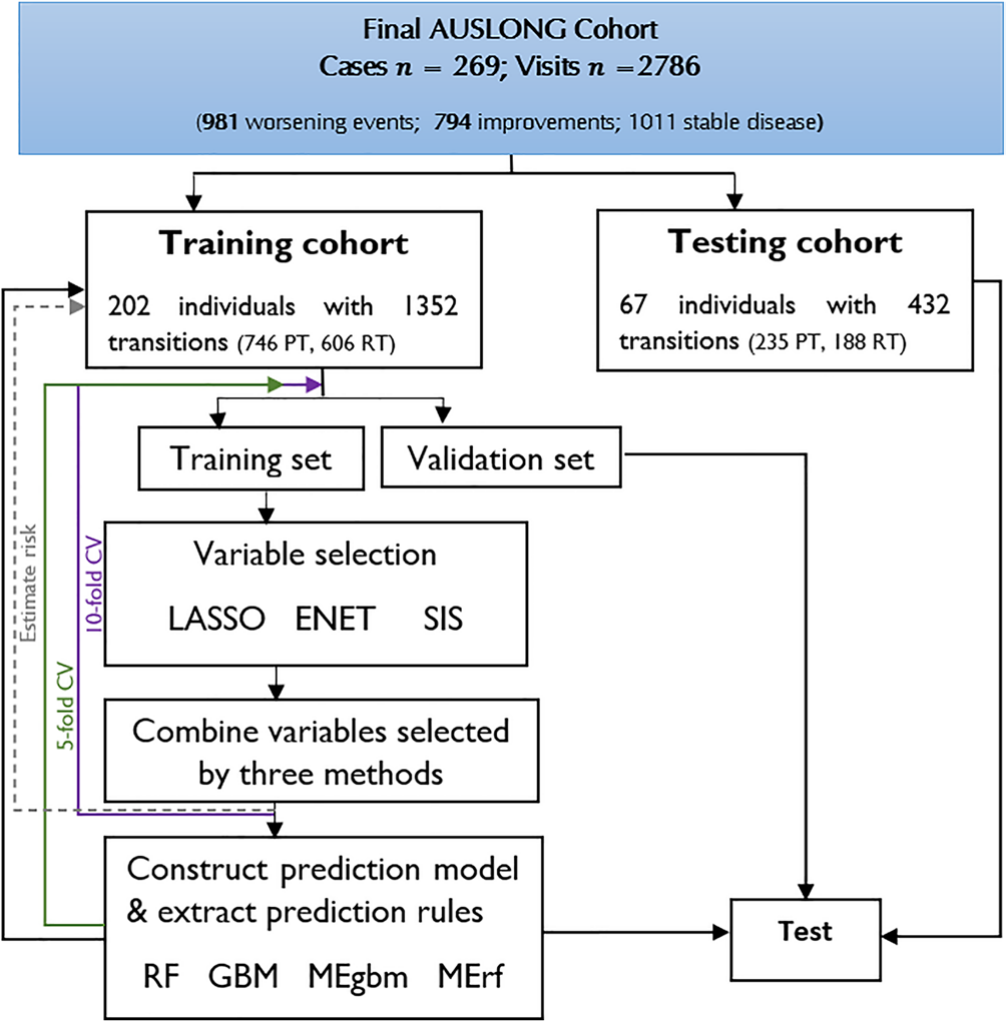
Outline of ensemble learning, and genetic risk prediction model construction. *PT* progressive transitions (“worsening” events); *RT* regressive transitions (“improved” events).

We randomly split the genotype data into *75%* training (*n* = 202), and *25%* test cohorts (*n* = 67) as depicted in Fig. 2. Utilising the training cohort, we first performed a global test to examine the added prognostic values of all SNPs ($n$ = 208) that passed the QC stage using the Goeman’s “globaltest” R-package^70^. Specifically, we tested the hypothesis (${H}_{0}: {\beta }_{1}={\beta }_{2}=,\dots ,{\beta }_{208}=0$ versus ${H}_{a}: {\beta }_{1}\ne {\beta }_{2}\ne ,\dots ,{\beta }_{208}\ne 0$) of no additional prognostic values of MS related genetic variants on the risk of worsening, conditional on the effects of clinical and environmental modifiers of disease (mentioned above). The significance level for this test was set to $p$ < 0.05^71^. Following the global test results, we applied three widely used penalised multivariable Cox models namely: least absolute shrinkage and a selection operator (*LASSO*), elastic net (*ENET*), and non-negative garrotte combined with sure independent screening (*NNG-SIS*), with tenfold cross-validation (CV) to select important SNPs. Because a SNP can affect one or multiple EDSS transition steps with effects in different directions, we added interactions with EDSSPREV. Utilising the training cohort, *LASSO* and *ENET* were fitted using the Goeman’s *penalised* R-package^72^, and *NNG-SIS* using customised survival functions^73^. SNPs having zero effect sizes were discarded.

#### Risk estimation and prognostic modelling

For the remaining SNPs selected across *LASSO*, *ENET* and *NNG-SIS*, a time-dependent multivariable Cox model with backward elimination (*α* = 0.05) was further employed to identify candidate SNPs^8^. This was achieved using the “mfp” R function^74^. Unbiased effect sizes for the candidate SNPs were then estimated in a random effects Cox model using the “*coxme*” R function^75^. In the Cox model, we posit that

2 $${\lambda }_{i}\left(t|x,b\right)={\lambda }_{0}\left(t\right)\mathrm{exp}\left({X}_{i}^{T}\beta +{\zeta }_{i}^{T}b\right),$$

$$b\sim N\left(0,\Sigma \left(\theta \right)\right),$$

where $\lambda \left(.\right)$ is the hazard function for the $i$th subject at time $t$, ${\lambda }_{0}$ is an unspecified baseline hazard function; ${X}_{i}^{T}$ is a fixed-effects design matrix containing SNPs dosages (including interactions with EDSSPREV), with a corresponding vector of fixed-effects regression coefficients $\beta$; ${\zeta }_{i}^{T}$ is a design matrix of random effects, with a corresponding vector of random effects estimates $b$; and $\theta$ is the variance of the random effects. Note that the random components are subject identifiers nested within MSTYPE.

### Stage 2: constructing genetic risk ensembles

To classify PwMS according to their disability status, we trained widely used RF and GBM models using the candidate genetic variants selected from stage 1 and compared their performance with MErf and MEgbm. The time-dynamic area under the receiver operating characteristics curve (AUC) was used to assessed model performance. This was achieved using internal and external fivefold cross-validation on the training cohort. That is, we split the entire training cohort into dynamic lagged training and internal validation sets (Fig. 2), such that predictors in the current visit were used to predict outcomes in the next visit^51^. The prediction model is a mixed-effects logistic model with random intercepts (subject identifiers nested within MSTYPE), and random slopes (random time effects). Next, mixed-effects logistics decision trees were constructed, and genetic decision rules were extracted using the *“inTrees”* (interpretable trees) algorithm in the *MEml* R-package^51^. To ensure non-redundant rule sets, we selected all rules of length between 2 and 5, with frequency < 0.01, and error < 0.25 in predicting future worsening events. In the mixed-effects logistic model used to classify MS subjects, we posit that

$${y}_{ij}\sim Bern\left({\mu }_{ij}\right), {y}_{ij}=\left\{\begin{array}{l}1, \quad if\, worsening\, status\\ 0, \quad otherwise \end{array}\right.,$$

3 $$\mathrm{logit}(\frac{{\mu }_{ij}}{1-{\mu }_{ij}})={(\beta }_{0}+{b}_{0i})+{X}_{i}^{T}\delta +{(b}_{1i}+{\beta }_{1}{t}_{ij}),$$

$${b}_{0i},{b}_{1i}\sim N\left(0,\Sigma \right), j=1,\dots {m}_{i}, i=1,\dots , N,$$

$$\sum_{i=1}^{N}{m}_{i}=2786; N=269,$$

where ${y}_{ij}$ is the event status for the $i$th subject at the $j$th time point; ${b}_{0i}$ are random deviations from the overall intercept (bias term) ${\beta }_{0}$; ${b}_{1i}$ are random deviations from the overall slope ${\beta }_{1}$, while $\Sigma$ is the variance–covariance matrix for the random effects. ${\beta }_{1}$ is the regression effect of the observation time (${t}_{ij}$) since diagnosis; ${X}_{i}^{T}$ is a fixed-effect design matrix containing SNP dosages (including interactions with EDSSPREV), with a corresponding vector of fixed-effects regression coefficients $\delta$.

### Stage 3: validation of the ensembles and their prediction rules

To evaluate the ensembles and the generated decision rules obtained in stage 2, we assessed externally the performance of the ensembles on the test cohort. Time-dynamic ROC (receiver operating characteristic curves) analysis was used to assess how well each model predicted future worsening events. The importance of the SNPs and their genetic decision rules in predicting future worsening of disability was estimated and evaluated on the training and test cohorts, respectively. We prioritised SNPs based on average Gini impurity and relative influence; and by their deleteriousness in the human genome using combined annotation dependent depletion (CADD) scores^76^.

### Functional annotation and gene enrichment analysis

Utilising the candidate prognostic variants, functional annotation and gene enrichment analyses were further conducted using the FUMA software as per the online manual^77^. The following parameters were used to further identify independent lead SNPs: maximum p-value for lead SNPs < 0.05, maximum p-value for annotation < 0.05, *r*^2^-threshold to define LD structure of lead SNPs ≥ 0.6, *MAF* ≥ 0.01, and maximum distance between LD blocks *d* < 250 kb. Because the raw p-values were derived from a multivariable analysis, *p* < 0.05 was used as the threshold cut-off.

## Results

We analysed a total of *269* FDE cases with *2786* EDSS transitions, with subsequent diagnosis as ROMS (n = 149), POMS (n = 12); SPMS (74), while 34 remained as CIS by the 10th year review. Of these, 76.8% (n = 205) were females, and the mean age at study entry was 37.5 years (SD = 9.9 years). Of the initial 279 cases (Fig. 1), 10 cases (seen once) were excluded from the analysis.

### Transition probabilities before and after EDSS imputation

The distribution of the transition percentages before and after EDSS imputation are shown in Table 1. We observed 11.4% (11.7%) transitions from the first state of disability (EDSS 0–1.5) into the second state of disability (EDSS 2–2.5) before (after) EDSS imputation, respectively. There were fewer transitions to and from extreme disability states (see zero entries on Table 1). A total of 516 transitions were made from state 1 (EDSS 0–1.5) into higher disability states. Additionally, PwMS were more likely to stay in a particular state of disability than to progress from it. For instance, the observed probability to stay in state 1 is 0.52, whereas the observed probability to stay in state 8 (EDSS 8–9.5) is 0.75. These probabilities were predicted a posteriori to be 0.40 and 0.50, respectively, after imputation. It is pertinent to note that the predicted posterior probabilities depend solely on the effects of clinical and environmental predictors (the “analysis variables”) included in the imputation model.

**Table 1:** EDSS transition percentages (%) before (after) imputation.

Number of MS subjects = 269, number of transitions before(after) imputation = 2029 (2786)									
To state									
From state	1	2	3	4	5	6	7	8	Total
1	51.7 (39.8)	11.4 (11.7)	14.9 (17.5)	18.6 (24.9)	2.7 (4.9)	0.6 (1.2)	0 (0)	0 (0)	516 (691)
2	13.3 (13.6)	40.5 (32.3)	15.0 (12.3)	25.1 (26.8)	4.9 (9.0)	1.4 (5.9)	0 (0)	0 (0)	346 (455)
3	18.8 (21.4)	12.8 (13.8)	44.6 (37.2)	17.0 (18.3)	5.4 (5.4)	1.2 (3.6)	0 (0)	0.3 (0.2)	336 (443)
4	14.4 (20.8)	15.1 (14.7)	12.4 (13.0)	46.0 (39.5)	9.6 (9.3)	2.4 (2.6)	0 (0)	0 (0)	450 (645)
5	7.7 (14.9)	8.8 (9.7)	6.6 (8.9)	14.4 (16.0)	48.9 (37.2)	12.6 (13.0)	0 (0)	0.5 (0.4)	182 (269)
6	0.5 (1.5)	0.5 (5.0)	0 (4.2)	5.9 (9.7)	8.1 (8.5)	81.7 (66.0)	1.6 (3.1)	1.6 (1.9)	186 (259)
7	0 (0)	0 (0)	0 (0)	0 (0)	0 (16.7)	80.0 (58.3)	20.0 (8.3)	0 (16.7)	5 (12)
8	0 (0)	0 (0)	0 (0)	0 (8.3)	0 (8.3)	0 (8.3)	25.0 (25.0)	75.0 (50.0)	8 (12)

The EDSS levels were categorised into disability states as: 1 = EDSS (0–1.5), 2 = EDSS (2–2.5), 3 = EDSS (3–3.5), 4 = EDSS (4–4.5), 5 = EDSS (5–5.5), 6 = EDSS (6–6.5), 7 = EDSS (7–7.5), 8 = EDSS (8–9.5).

Zero entries on the table represents rare transitions.

Transitions at lower disability states (1, 2, 3, 4 & 5) were more frequent than transitions at higher levels of disability (6, 7, & 8). Additionally, previous disability states (EDSSPREV) were key determinants of future states, and thus satisfies the first-order Markov’s process described above (see Web Appendix A1). Overall, all variables in the imputation model were imputed with high accuracy judging from Gelman-Rubin’s diagnostic criteria (GR-crit ≤ 1.1, Web Appendix A1), the density plots and the mixing rates of the Markov chains (Web Appendix A2).

### Identification and annotation of candidate genetic effects

**Figure 3:**
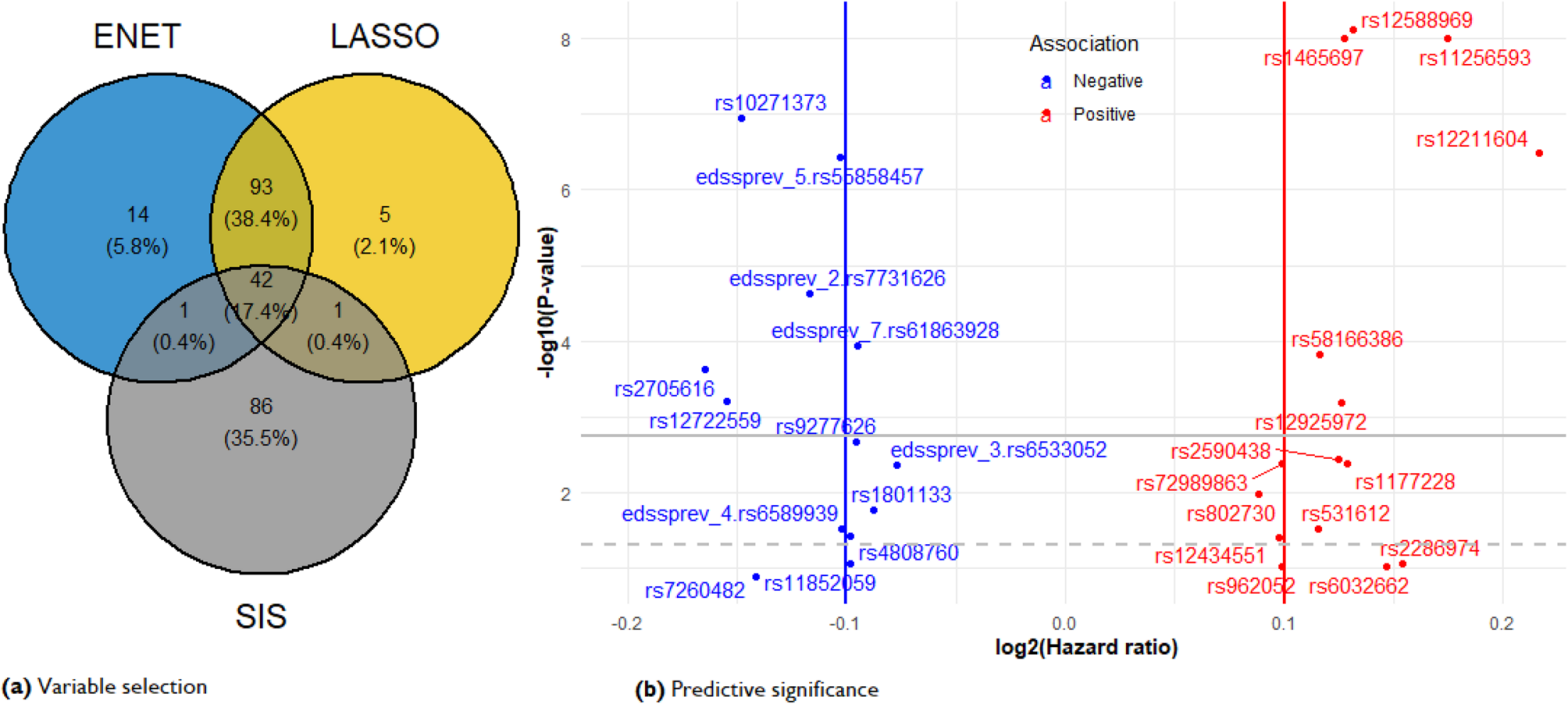
(**a**) Number of genetic variations shared among tenfold CV by variable selection methods. (**b**) Volcano plot significance of SNPs in the multivariate prognostic model. Statistical significance at the multivariate cut-off level is − log10($\alpha$ = 0.05) = 1.30 (grey dash-lines), family-wise error rate at − log10($\alpha$ = 0.05/28) = 2.75 (grey solid-lines).

After quality control, *208* of the *232* list of MS risk loci from the IMSGC (10.1126/science.aav7188?url_ver=Z39.88-2003&rfr_id=ori:rid:crossref.org&rfr_dat=cr_pub%20%200pubmed)^39,60^ (including *rs3129889* that tags HLA-DRB1*1501 genotype) were extracted from our AusLong database. The global test for the null hypothesis of no additional prognostic values of all 208 genetic variants given the effects of the clinical and environment predictors was rejected (*Z-score* = 0.212, *p* = 7.95 × 10^–15^). Following this result, all 208 SNPs were included in stage 1. The number of genetic variants that resulted from the screening methods is presented in Fig. 3a. A total of 147 genetic variants (including interactions with EDSSPREV) were selected across *LASSO, ENET*, and *NNG-SIS*, respectively. Notably, *LASSO* and *ENET* produced very similar results within tenfold CV on the training cohort, whereas *NNG-SIS* identified 86 unique associations. Of the 147 genetic variants (Fig. 3a) selected across the penalised Cox models, 28 candidates (*p* ≤ 0.05) were retained in the final prognostic model. Positional and *eQTL* mapping revealed that these SNPs were in close proximity to *33* unique genes. We obtained identical results with (without) imputation of EDSS, respectively. However, the imputed model performed slightly better (AIC = 10,271.97) than the model without EDSS imputation (AIC = 10,272.26, Web Appendix C3). Table 2 shows results for the imputed model obtained with 28 candidate variants.

**Table 2:** A genetic ensemble model for predicting disability progression in multiple sclerosis.

SNP	CHR	POS	Alleles	Region	Nearest Gene	P-value	HR	$\upbeta$	SE	avIMP	CADD
$rs{61863928}^{{{\varvec{\gamma}}}_{7}}$	10	64,449,549	G/T	Exon	*ADO*	1.2e−04	0.94	− 0.07	0.02	1.00	15.36
$rs12722559$	10	6,070,273	C/A	Upstream	*IL2RA*	6.1e−04	0.90	− 0.11	0.03	0.94	6.50
$rs4808760$	19	18,301,979	G/C	Upstream	*IL12RB1*	3.8e−02	0.93	− 0.07	0.03	0.86	8.82
$rs9277626$	6	33,081,823	G/A	Exon	*DPB2*	2.2e−03	0.94	− 0.07	0.02	0.85	9.03
$rs12434551$	14	69,253,364	A/T	Exon	*ZFP36L1*	4.0e−02	1.07	0.07	0.03	0.85	2.39
$rs7260482$	19	45,143,942	A/C	Exon	*PVR*	1.3e−01	0.91	− 0.10	0.06	0.85	1.15
$rs12588969$	14	103,230,758	C/G	Exon	*RCOR1*	2.1e−10	1.10	0.09	0.01	0.84	15.09
$rs6032662$	20	44,734,310	C/T	Exon	*SLC12A5*	9.3e−02	1.11	0.10	0.06	0.84	5.67
$rs11256593$	10	6,117,322	T/C	Upstream	*IL15RA*	5.1e−57	1.13	0.12	0.01	0.84	1.28
$rs802730$	6	128,280,104	T/C	Exon	*THEMIS*	1.1e−02	1.06	0.06	0.02	0.83	11.86
$rs962052$	2	151,644,203	C/T	Exon	*RBM43*	9.7e−02	1.07	0.07	0.04	0.83	3.37
$rs1465697$	19	49,837,246	C/T	Upstream	*DKKL1*	1.7e−128	1.09	0.09	0.00	0.83	2.10
$rs2590438$	3	187,565,968	T/G	Exon	*BCL6*	3.6e−03	1.09	0.09	0.03	0.83	1.45
$rs1801133$	1	11,856,378	A/G	Missense	*MTHFR*	1.7e−02	0.94	− 0.06	0.03	0.80	25.6
$rs11852059$	14	52,306,091	A/C	Upstream	*FRMD6*	8.8e−02	0.93	− 0.07	0.04	0.80	5.06
$rs531612$	11	65,705,432	C/T	Exon	*EHBP1L1*	3.0e−02	1.08	0.08	0.04	0.80	0.13
$rs12925972$	16	79,111,297	C/T	Intron	*DYNLRB2*	6.6e−04	1.09	0.09	0.03	0.79	8.26
$rs1177228$	2	61,242,410	G/A	Upstream	*PUS10*	4.1e−03	1.09	0.09	0.03	0.79	0.08
$rs2286974$	16	11,114,512	G/A	Exon	*CLEC16A*	8.5e−02	1.11	0.11	0.06	0.77	0.43
$rs2705616$	4	87,862,396	G/C	Intron	*AFF1*	2.3e−04	0.89	− 0.11	0.03	0.76	3.36
$rs58166386$	19	16,559,421	G/A	Intron	*EPS15R*	1.5e−04	1.08	0.08	0.02	0.75	0.14
$rs10271373$	7	138,729,795	C/A	UTR-3	*ZC3HAV1*	1.1e−07	0.90	− 0.10	0.02	0.74	10.90
$rs72989863$	4	164,493,807	G/A	Intron	*MARCH1*	4.1e−03	1.07	0.07	0.02	0.73	0.24
$rs{55858457}^{{{\varvec{\gamma}}}_{5}}$	7	2,443,302	G/T	Upstream	*CHST12*	3.7e−07	0.93	− 0.07	0.01	0.71	2.06
$rs12211604$	6	7,100,029	A/G	Upstream	*RREB1*	3.2e−07	1.16	0.15	0.03	0.63	0.05
$rs{6533052}^{{{\varvec{\gamma}}}_{3}}$	4	103,911,781	A/G	Upstream	*SLC9B1*	4.4e−03	0.95	− 0.05	0.02	0.62	2.12
$rs{7731626}^{{{\varvec{\gamma}}}_{2}}$	5	55,444,683	G/A	Intron	*ANKRD55*	2.4e−05	0.92	− 0.08	0.02	0.50	1.37
$rs{6589939}^{{{\varvec{\gamma}}}_{4}}$	11	122,518,525	A/G	Intron	*UBASH3B*	3.0e−02	0.93	− 0.07	0.03	0.41	1.39

Transition-specific SNPs have superscripts ${{\varvec{\upgamma}}}_{\left(.\right)}$ indicating their interaction with previous EDSS levels (EDSSPREV).

The disability states based on previous EDSS levels are define by the parameters:

${\upgamma }_{1}:$ state = 1;${\upgamma }_{2}:$state = 2; ${\upgamma }_{3}:$state = 3,${\upgamma }_{4}:$state = 4,

${\upgamma }_{5}:$stage = 5,${\upgamma }_{6}:$state = 6,${\upgamma }_{7}:$state = 7,${\upgamma }_{8}:$state = 8.

The volcano plot (Fig. 3b) reveals *12* SNPs having *p*-values below the family-wise threshold (*p* = 0.002). Most of these had minor allele frequencies > 10%. We observed 73% and 63% differences in individual progression rates, and progression rates due to MSTYPE, respectively (intra-class correlations). The proportion of total liability attributable to the 28 candidate variants at the individual level was 0.47. Further, the proportion of total liabilities conditional on MSTYPE were: 0.49 (CIS), 0.43 (ROMS), 0.36 (SPMS), and 0.42 (POMS). Note that MSTYPE liability estimates can be influenced by group size. Functional annotation and gene enrichment analysis using FUMA software^77^ revealed *7* lead SNPs (*rs12211604*, *rs7731626*, *rs55858457*, *rs10271373*, *rs11256593*, *rs12588969*, *rs1465697*), some of which were located near, or tagged to one of *13* genes enriched in peptide hormones and steroids biosynthesis, respectively (Web Appendix B).

### Interpretation of the validated ensembles

**Figure 4:**
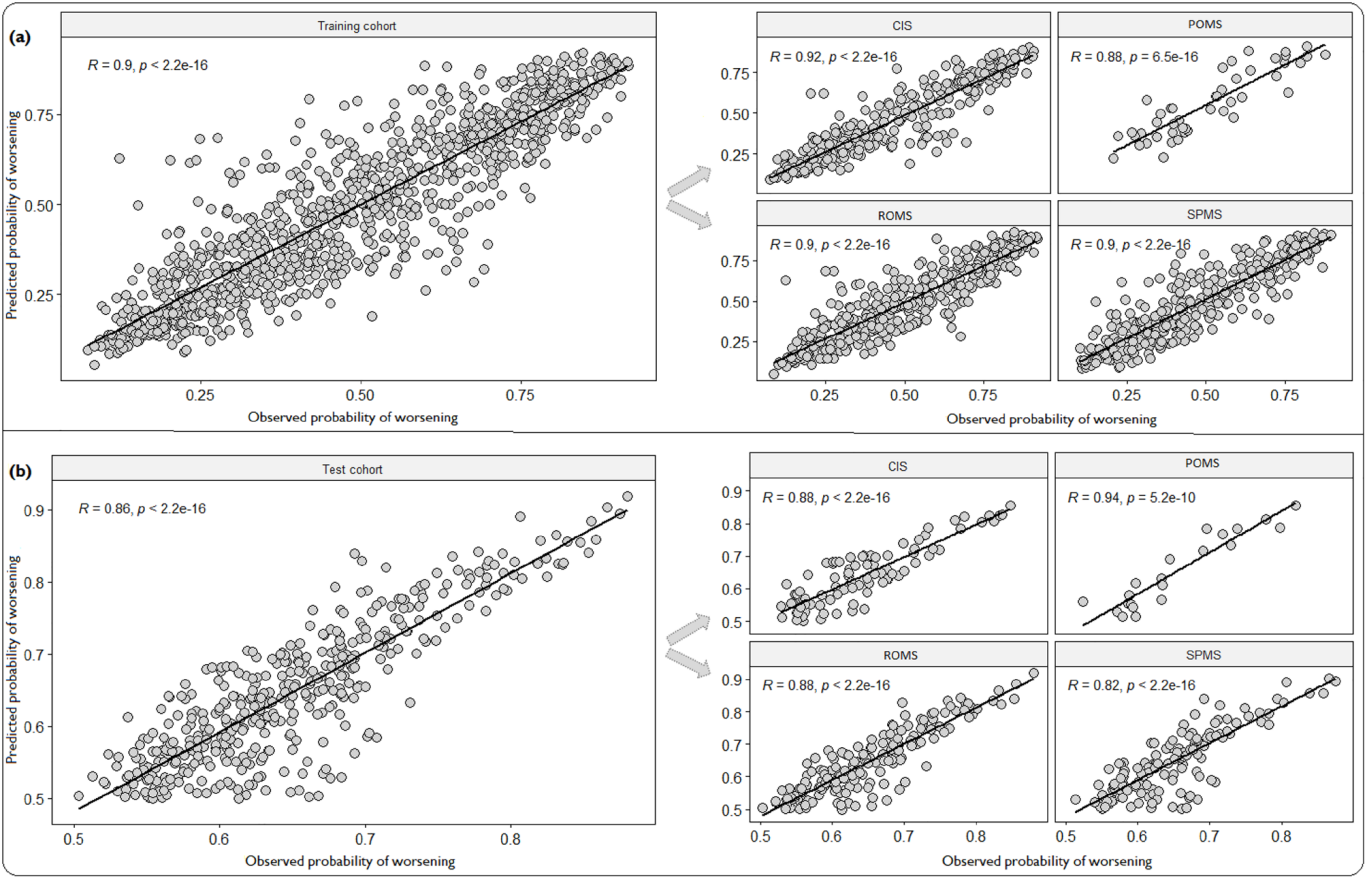
Predicting future disability progression. Correlation between the observed and predicted probability of worsening events stratified by MS phenotype in the training (**a**) and test cohort (**b**).

**Figure 5:**
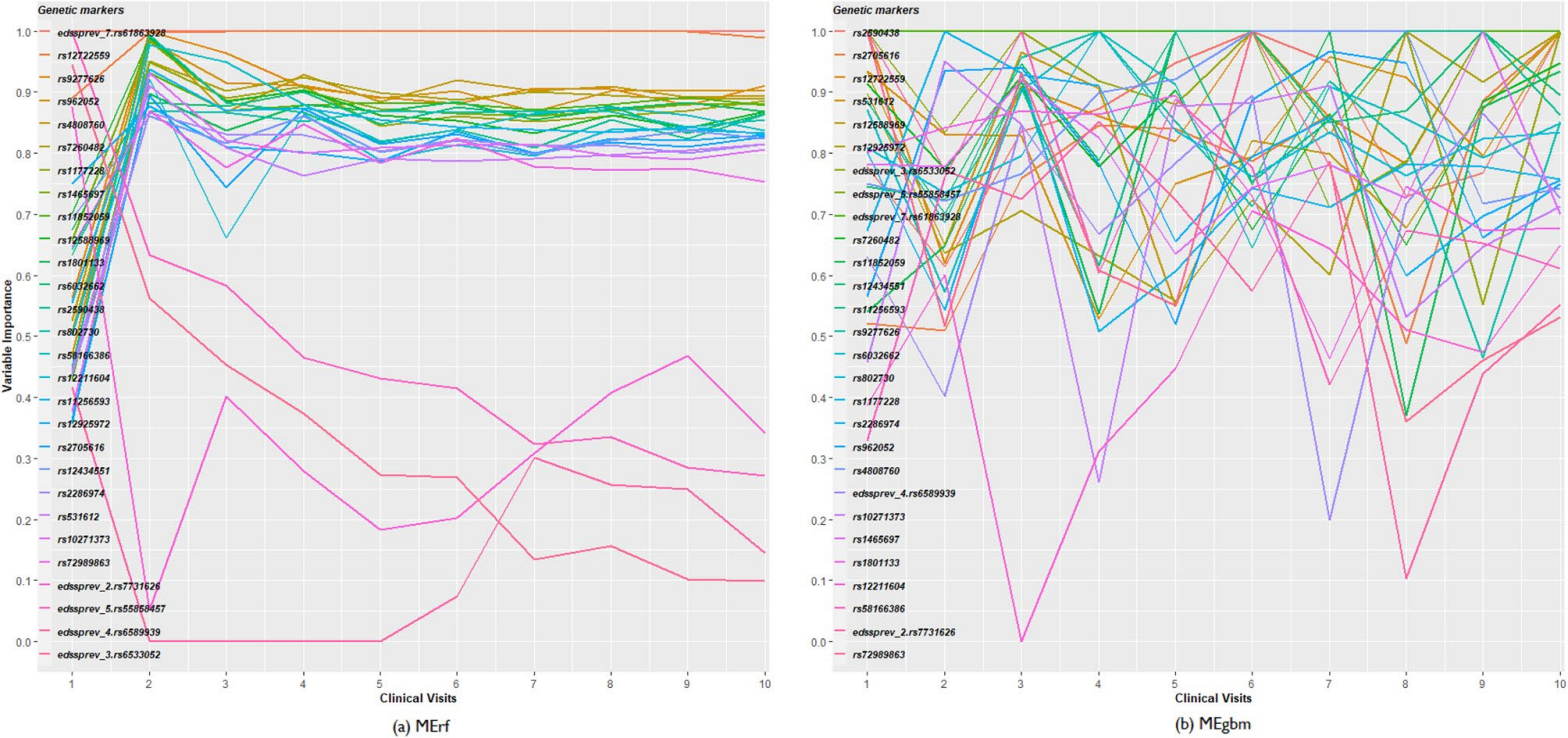
Ranking of genetic markers by 2 methods (**a**) MErf and (**b**) MEgbm. These curves shows the relative importance of genetic variants in predicting worsening of disability over time. Each line on the plot is a genetic marker. The color of the lines matches the color of the genetic variants. From left to right, the importance of a genetic marker in predicting worsening events changes over time (clinical visits).

MErf and MEgbm had the highest accuracies in the training and testing cohorts (Web Appendix C1) and are best suited to describing subject characteristics that influence disability progression over time. Particularly, as the number of repeated observations increased with time, we observed better performance using MErf and MEgbm (Web Appendix C2), whereas the performance of RF and GBM deteriorated. Although all ensembles used identical marker sets, the increased performance observed with MErf and MEgbm indicated that these methods take advantage of the increasing sample size and correlation induced by multiple observations within a subject to yield more robust models. Overall, the ensemble-derived predicted disability worsening outcomes correlated well with the observed outcomes in both the training (*r* = 0.90, *p* = 2.2 × 10^–16^) and testing (*r* = 0.86*, p* = 2.2 × 10^–16^) cohorts, respectively (Fig. 4). Additionally, we found consistent results conditional on MS disease course phenotype (Fig. 4). Figure 5 shows the relative importance scores (scaled 0 to 1) in predicting disability, with significant changes observed over time. Notably, none of the top 7 SNPS have been shown to have a functional role in MS disability accrual, although likely to have plausible biological effects.

### Interpretation of genetic decision rules

**Figure 6:**
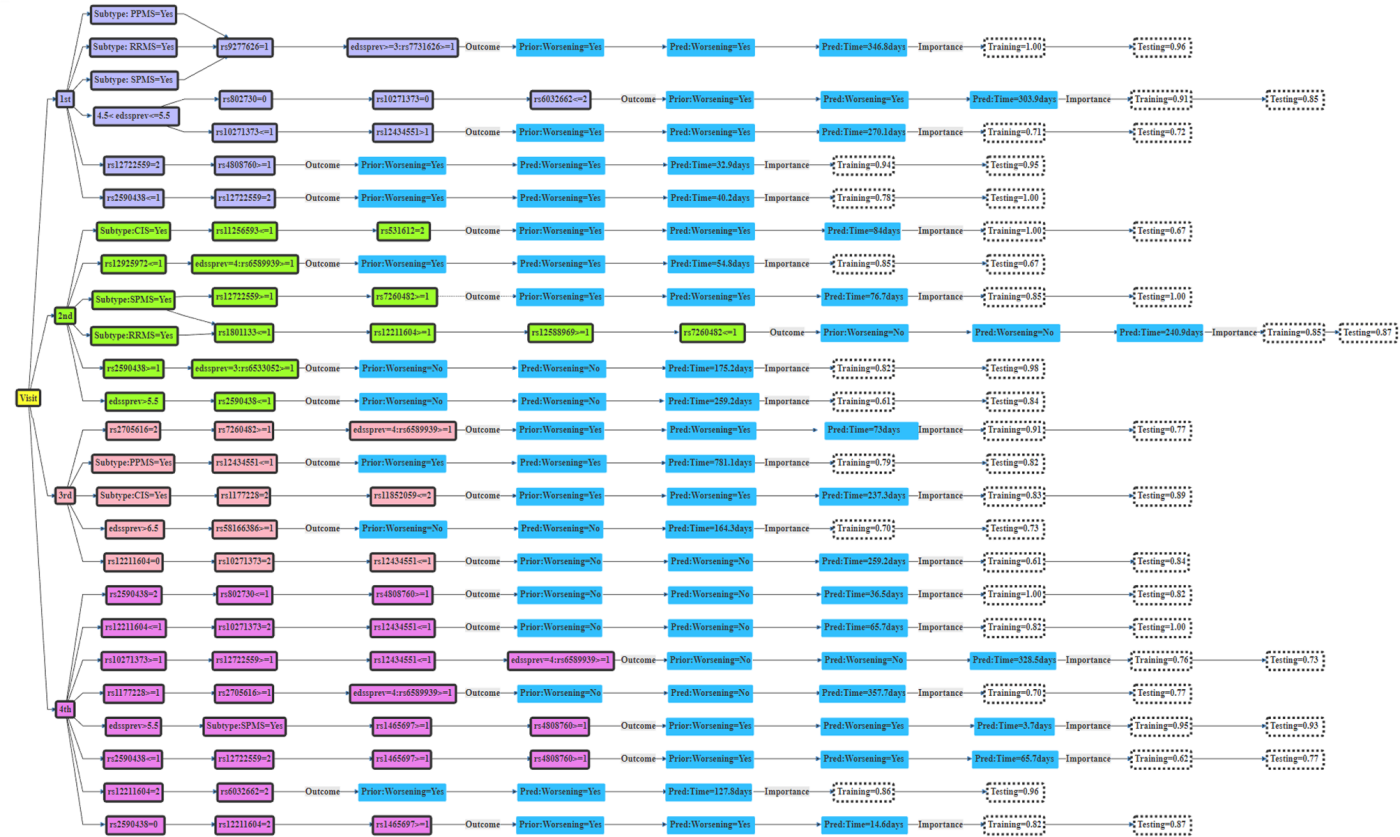
Genetic decision rules for predicting future worsening of disability for PwMS. These rules indicate how the MErf ensemble model combines allele dosages from a set of influential genetic variants amongst the 28 candidates SNPs to make prediction decisions regarding future disability. Each rule indicates the expected EDSS transition time conditional on the effects of genetic variants, MS disease course (MSTYPE), and previous disability histories (EDSSPREV). Only rules for the first four visits have been shown.

MEgbm and MErf ensembles produced identical sets of decision rules constructed using the 28 SNP candidates. Figure 6 shows a decision tree of the top genetic decision rules extracted from both methods (shown for the first 4 visits). The importance of these rules in the training and testing cohorts are shown at the end of the leafs at each visit. These rules indicate how frequent the individual ensembles decision trees combine a set of influential genetic variants among the 28 candidates to make prediction decisions regarding future disability status for a person living with MS. For instance, during the first clinical visit, the MErf ensemble uses *2* (*rs9277626* and *rs7731626*) of the *28* SNPs candidates to correctly classify subjects of different MS subtypes in the training cohort prone to future worsening of disability (score = 100%), given that they were previously in state 2 (EDSS 2–2.5). The average time to transition from state 2 into higher disability states was 346.8 days. This rule was applicable to 96% of MS subjects in the testing cohort (Fig. 6).

## Discussion

In this study, we identified 28 significant MS genetic loci associated with risk of worsening of disability over time. We showed that these loci had additional prognostic values when combined with clinical and environmental predictors. To predict disability worsening outcomes, we developed and validated simple, learnable, interpretable, and robust ensemble genetic machine learning models. Using the derived ensembles, genetic decision rules were constructed to identify PwMS prone to future worsening of disability. Future disability states were significantly influenced by prior disability histories. Additionally, we showed that the derived ensembles, especially MEgbm and MErf, had better sensitivities and accuracies in predicting worsening outcomes over time. Despite these findings, there is little current knowledge on the functional implications of the identified associations.

Different estimates of the variance in disability progression explained by MS related genetic variants have been reported in previous studies^33,40,41,43,78–80^. For instance, using 125 early MS cases with *5* years of follow-up from our cohort, Pan et al*.*^41^, constructed a genetic risk score from 7 of 116 MS risk variants^39,60^ to explain 32.7% of the variance in annualised EDSS, but did not validate their findings externally; whereas Jackson et al*.*^40^, developed a RF-based genetic model on MS disease severity scores (MSSS) which included 19 of ~ 200 autosomal SNPs^39,60^ to explain 21% of the variability in MSSS, with just 4% chance of validating their results externally. However, it is unclear how these models make prediction decisions and/or account for correlation induced by repeated EDSS measurements within a subject. Moreover, the AUC used to assess the performance of these models was fixed rather than time-dynamic, as would be expected given the dynamic nature of EDSS transitions. Therefore, a common drawback to these studies is not the variability explained, but rather the utility and reliability of the identified associations and the derived predictions in clinical practice.

In our study, we made a considered and clinically plausible Markov’s assumption (i.e., that future disability is predicated on the prior disability history) to study MS disability progression process in continuous time. We employed robust MEML ensembles to predict future disability worsening outcomes. Of the 28 common MS risk loci identified, 7 were independent non-functional SNPs having the greatest effects on worsening outcomes. However as with MS risk, it is very difficult to provide actual biological mechanisms for the identified SNPs associations, other than just non-specific genetic markers of disability progression. For instance, the SNP *rs12211604* is located on chromosome 6, upstream of the promoter region of the *RREB1* gene. The *RREB1* gene is widely involved in biological processes including cell proliferation, transcriptional regulation, and DNA damage repair^51^. Specifically, it encodes a zinc finger transcription factor that binds to RAS-responsive elements (RREs) on the calcitonin gene promoter, thereby increasing calcitonin expression^81,82^. In order to ensure proper nerve cell function, and smooth muscle contractions, the calcitonin hormone lowers blood calcium levels^48^. However the effect of the *rs12211604* variant on *RREB1* gene expression levels has not been investigated to date.

Instead of relying on the complex predictions generated by the MEML ensembles to make prediction decisions, here we presented simple, readable, and transparent relational rules sets that could be translated to aid existing clinical predictions^21,44,48,50^, or clinical research studies. This can be achieved via a web application delivering equal prediction accuracy as the original ensemble. Clinicians could use these rules (provided genotyping was available) alongside recent clinical predictions^21,44,48,50^, and identify PwMS at greater risk of disability accrual in the short and medium term, and institute more aggressive MS therapies where indicated^25^. For instance, during the first clinical visit (Fig. 6), a person with MS having *2* alleles for *rs12722559* and ≥ 1 allele for *rs4808760* has a faster rate of disability accrual (expected time of transitioning is ~ 33 days), compared to some one having ≤ 1 allele for *rs10271373* and > 1 allele for *rs12434551* (expected time of transitioning is 270 days). Further, incorporating recently established clinical biomarkers such as brain MRI T2L load, baseline blood CSF parameters^18,19^ and NFL^18,20^; and disease modifiable risk factors such as VitD treatments and type of DMT use^7–10^, will enhance the clinical utility of these decision rules. Additionally, combining the effects of the 28 variants into a standard polygenic risk score (PRS) may further improve the predictive accuracy of the derived ensembles. However, it is important to note that prognostic decisions based on PRS will lead to loss of information and interpretation^83,84^ of the individual SNP-based genetic decision rules.

The strengths of this study lies in the assumptions we made regarding the underlying disability process in MS (defined above), and the use of novel machine learning platforms capable of analysing the longitudinal changes in EDSS scores. By analysing the continuous-time evolution of EDSS transitions, the total genetic liability in progression rates attributable to the 28 candidate variants was substantially increased compared other studies^24,31–36,40,41,43,80^. In particular, the high intra-class correlations between the observed and ensemble-derived predicted probabilities of worsening revealed a good fit to the model. The obtained p-values for these correlations (all *p* $\le$ 5.2 × 10^–10^) were far smaller than recently reported^40,80^, suggesting a near 100% chance of replicating our results in an external MS cohort.

Nevertheless, we recognise limitations in our study. For instance, our genetic ensemble lacks genome-wide coverage, and epistatic interactions amongst the MS genetic loci used. A genome-wide analysis to further identified novel SNPs associations which are not MS related**,** could be a fruitful area of future research. Similarly, we lack an external validation cohort (an external MS population) that matches our prospective, data dense AusLong cohort with genotyping available. Thirdly, as emphasised, the genetic variants utilised here do not have any established biological effect making it difficult to elucidate the actual mechanisms underlying MS progression from these data.

In conclusion, our study provides a simple, learnable, interpretable, and robust ensemble genetic machine learning model(s) that aggregates association evidence from 28 candidate MS risk loci to predict future worsening of disability in PwMS. Our ensembles provided genetic decision rules which could be translated to provide additional prognostic values to existing clinical prediction models^21,44,48,50^, with the additional benefit of incorporating relevant genetic information into clinical decision making for PwMS. Finally, modeling the continuous-time evolution of EDSS increased the variance in disability progression that is genetically determined.

## Supplementary Information

Supplementary Information.

## Data Availability

The Auslong SNP genotype datasets generated and/or analysed during the current study are available in dbGaP under study Accession: phs000139.v1.p1. Direct access to the Auslong phenotype data can be obtained from the AusLong Investigators Research Group (https://www.msaustralia.org.au/ausimmune/) through the corresponding authors BVT.
