# Supplementary material for: Ensemble machine learning identifies genetic loci associated with future worsening of disability in people with multiple sclerosis
Source: Sci Rep. 2022 Nov 11;12:19291. doi: 10.1038/s41598-022-23685-w (PMC9652373; doi:10.1038/s41598-022-23685-w)
Supplement: Supplementary file 1 — Supplementary Information. [file 41598_2022_23685_MOESM1_ESM.pdf]

Web-based Supplementary Material for:
Ensemble machine learning identifies genetic loci associated with future worsening of disability in people with Multiple Sclerosis.

Valery Fuh-NGWA
Valeryfuh.ngwa@utas.edu.au

October 10, 2022

1 Appendix A1: Imputation of EDSS

```
#################################################################
    Bayesian cumulative logit mixed model fitted with JointAI
#################################################################

## R-Code: For imputation of EDSS

library(JointAI)
Call:
clmm_imp(fixed = edss ~ sex + age + ebv + bmi + smoker + hads +
    edssprev_2 + edssprev_3 + edssprev_4 + edssprev_5 + edssprev_6 +
    edssprev_7 + edssprev_8, data = msdat, random = ~1 | id,
    n.chains = 3, n.adapt = 5000, n.iter = 10000, thin = 5,
    monitor_params = c(imps = TRUE,analysis_main = TRUE),
    auxvars = ~latexp + vitd + mstype,
    refcats = "first")


Posterior summary:
               Mean      SD     2.5%   97.5%  P-value  GR-crit   MCE/SD
sex[Female]  -0.1586 0.20404 -0.55150  0.2401   0.429    1.02    0.0541
EBNA          0.1526 0.14254 -0.12336  0.4328   0.278    1.00    0.0147
Age           0.0583 0.00735  0.04415  0.0729   0.000    1.01    0.0246
BMI           0.0174 0.01236 -0.00734  0.0418   0.157    1.00    0.0221
smoker[Yes]  -0.0786 0.17188 -0.41429  0.2655   0.638    1.00    0.0279
hads          0.0699 0.01072  0.04896  0.0911   0.000    1.00    0.0205
edssprev_2    1.1323 0.12402  0.89331  1.3775   0.000    1.00    0.0163
edssprev_3    0.9975 0.12923  0.74124  1.2467   0.000    1.01    0.0177
edssprev_4    1.3569 0.12474  1.11373  1.6023   0.000    1.00    0.0190
edssprev_5    2.5623 0.19161  2.18753  2.9399   0.000    1.00    0.0173
edssprev_6    4.6562 0.29057  4.10134  5.2345   0.000    1.00    0.0195
edssprev_7    4.8539 1.16908  2.49932  7.1638   0.000    1.00    0.0161
edssprev_8    9.8487 1.20807  7.56750 12.3206   0.000    1.00    0.0142

Posterior summary of the intercepts:
            Mean    SD    2.5%  97.5% tail-prob. GR-crit MCE/SD
edss > 1   0.612 0.223   0.171  1.047    0.00567    1.02 0.0547
edss > 2  -0.553 0.223  -0.991 -0.121    0.01100    1.03 0.0517
edss > 3  -1.562 0.225  -2.006 -1.128    0.00000    1.03 0.0516
edss > 4  -3.444 0.236  -3.925 -2.989    0.00000    1.03 0.0407
```

```
edss > 5   -5.084 0.258  -5.599 -4.592    0.00000     1.02 0.0439
edss > 6   -9.978 0.447 -10.854 -9.114    0.00000     1.01 0.0262
edss > 7 -11.044 0.555 -12.177 -9.983    0.00000     1.01 0.0237

Posterior summary of random effects covariance matrix:
               Mean    SD  2.5% 97.5% tail-prob. GR-crit MCE/SD
D_edss_id[1,1] 1.32 0.239 0.909  1.84                  1  0.022


MCMC settings:
Iterations = 5005:15000
Sample size per chain = 2000
Thinning interval = 5
Number of chains = 3

Number of observations: 3065
Number of groups:
 - id: 279
```

2 Appendix A2: Assessing the quality of the Imputation model

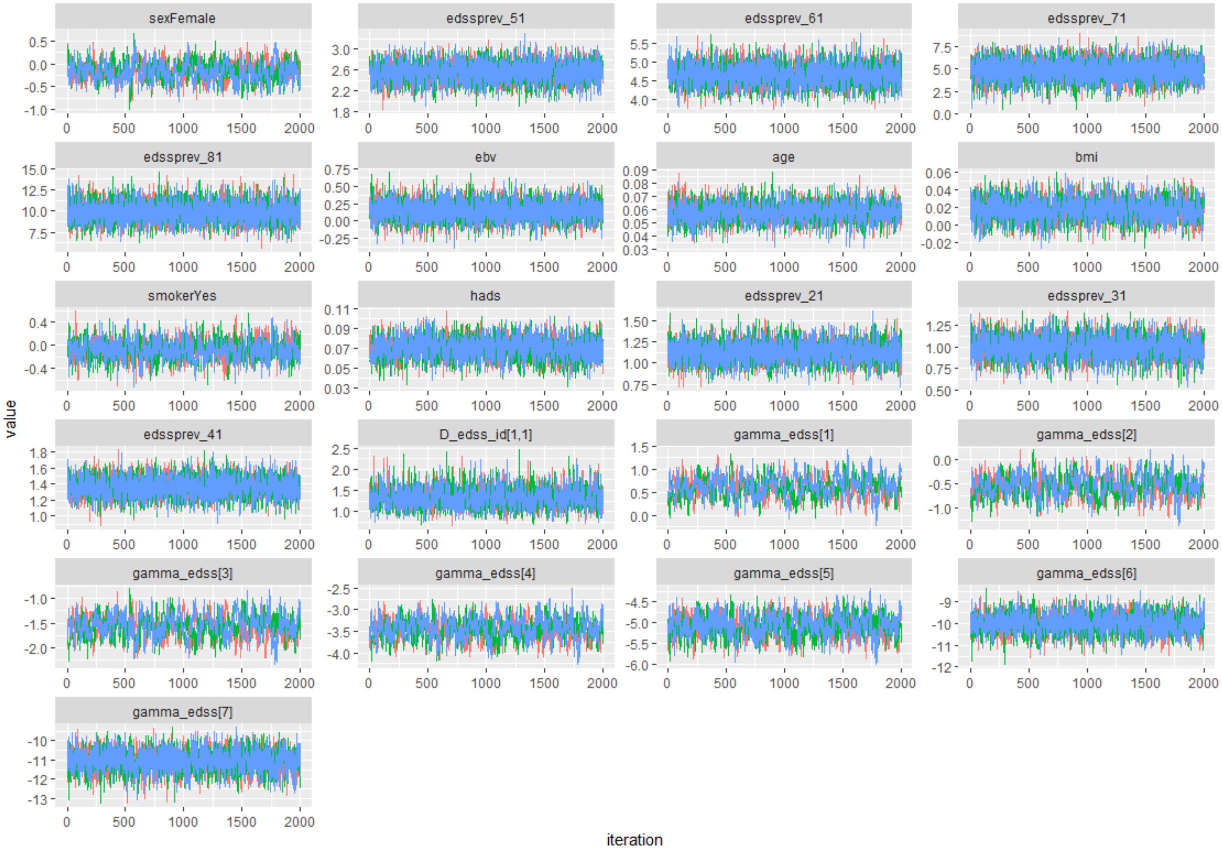

Figure 1: A trace plot of the main analysis variables indicating a good mixing rate of the Markov Chains

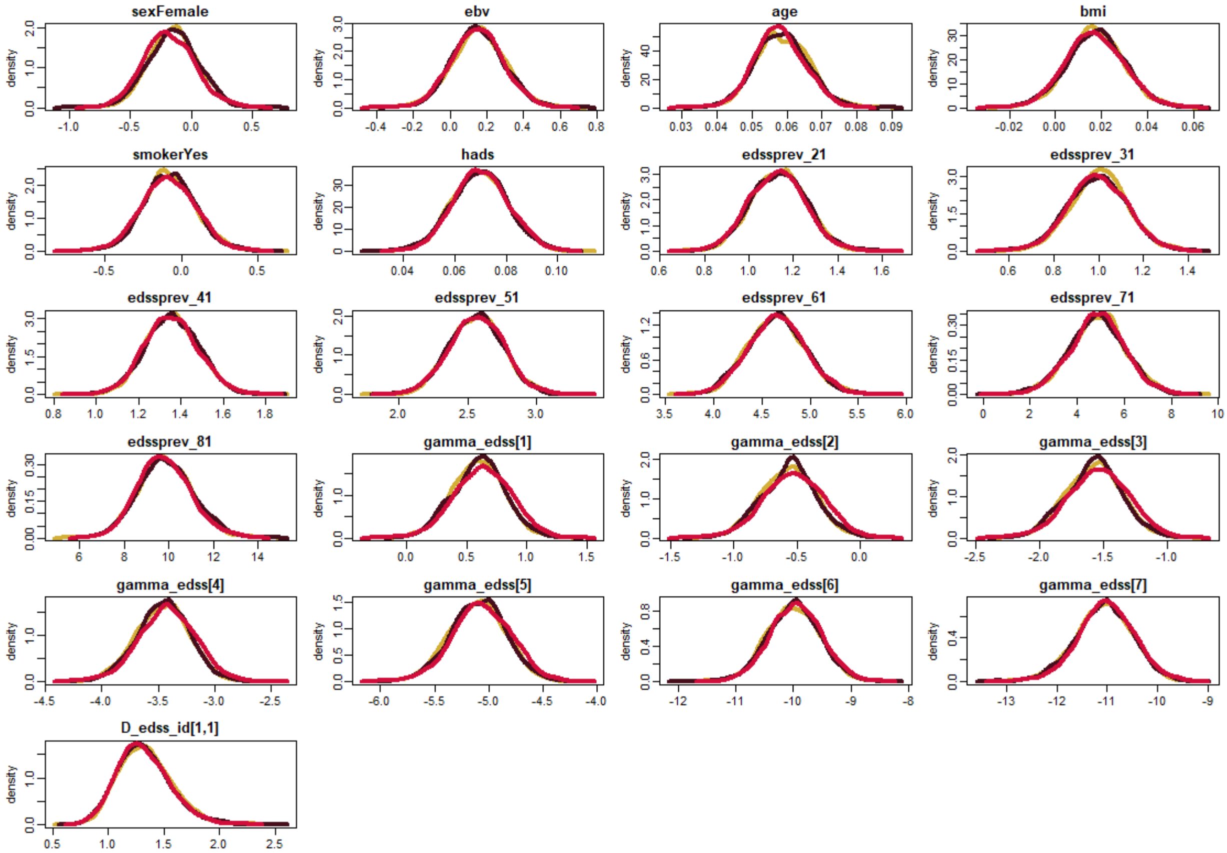

Figure 2: A density plot of main analysis variables showing satisfaction of the normality assumption for the regression parameters

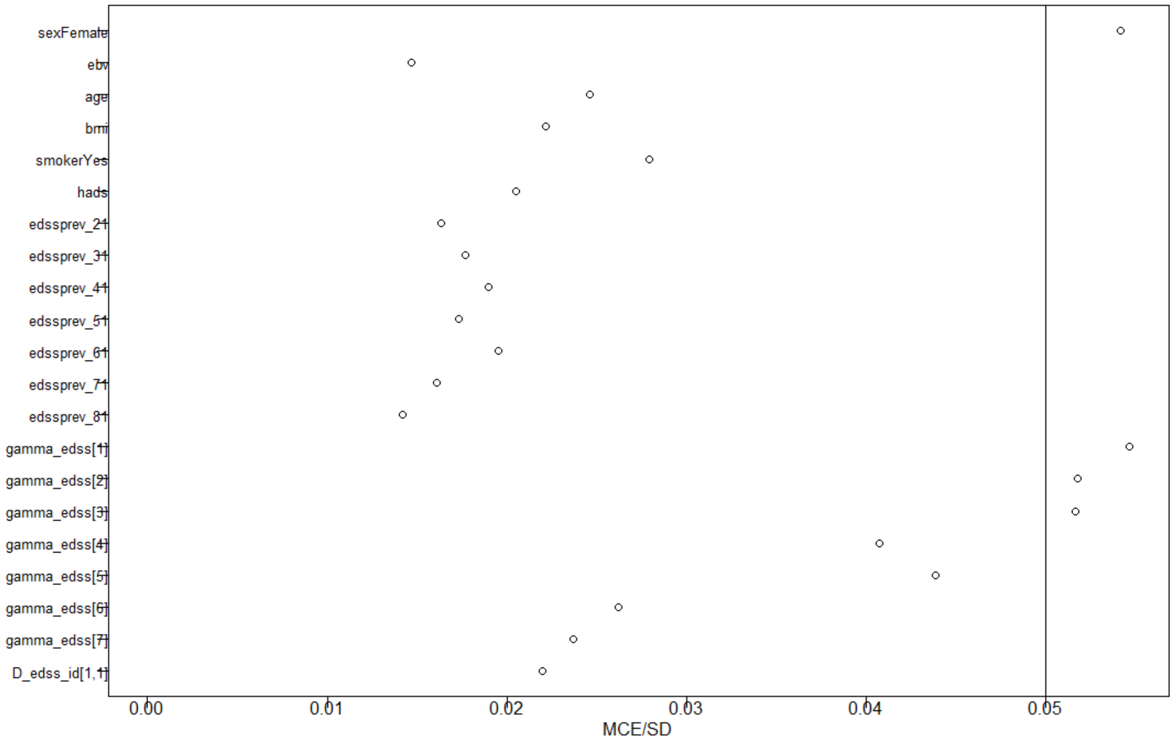

Figure 3: Monte Carlo standard errors for most parameters are below the 5% margin

3 Appendix B: Functional Analysis of the identified Associations

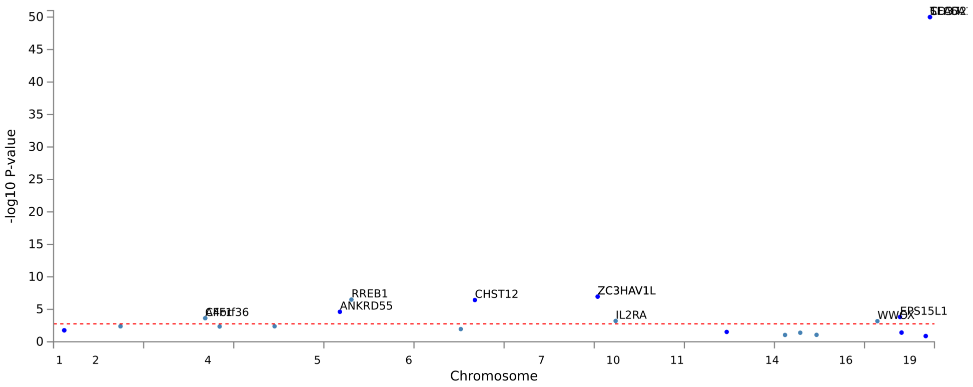

Figure 4: A manhattan plot of the gene-based test computed by MAGMA using SNP-outcome association summary statistics. Input SNPs were mapped to 13 protein coding genes. Genome wide significance (red dashed line in the plot) was defined at P=-log10(0.05/28)=2.748188

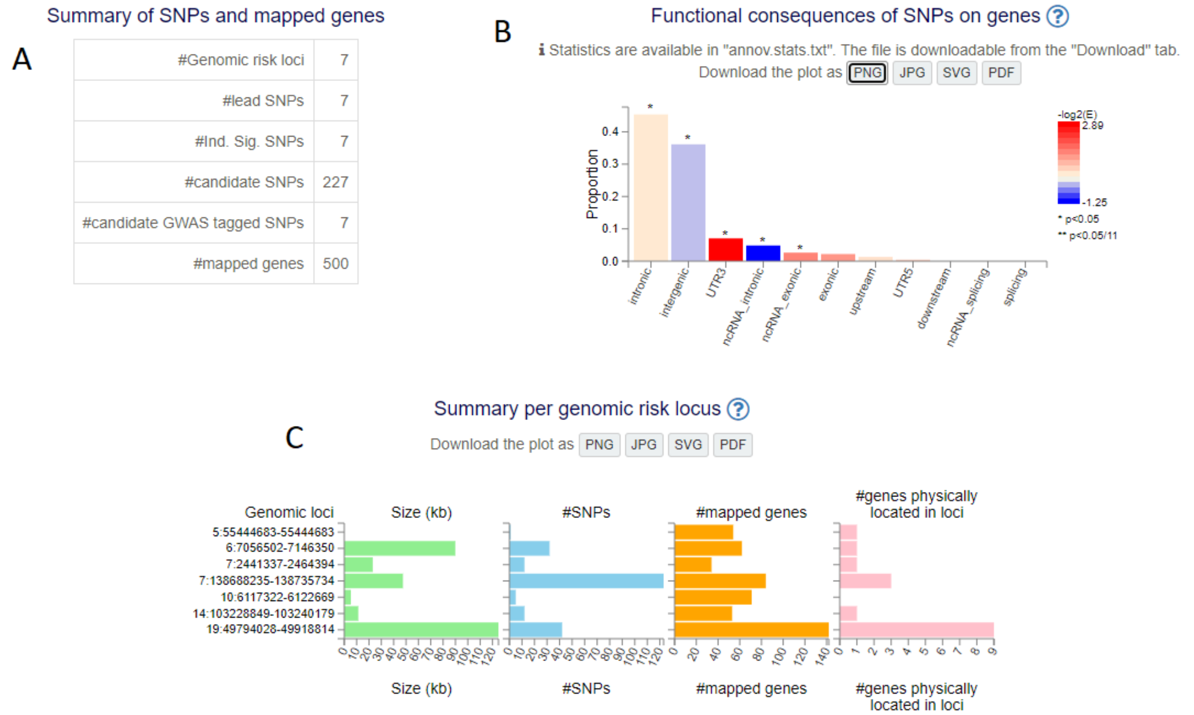

Figure 5: Functional NSP mapping and gene enrichment analysis. **Panel A**: Summary of independent lead SNPs and mapped genes. **Panel B**: Functional consequences of lead SNPs on genes. The histogram displays the proportion of SNPs (all SNPs in LD of independent significant SNPs) which have corresponding functional annotation assigned by ANNOVAR. Bars are colored by log2(enrichment) relative to all SNPs selected in the reference panel. **Panel C**: Total number of SNps per genomic locus. The histograms displays summary results per genomic locus. Note that the genomic loci can contain more than 1 independent SNPs

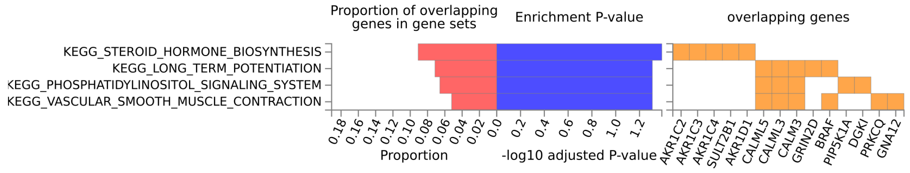

Figure 6: Functional enrichment of input genes in Gene Sets using the Kyoto Encyclopedia of Genes and Genomes (KEGG). Shown are the pathways/targets in which the enriched genes are involved

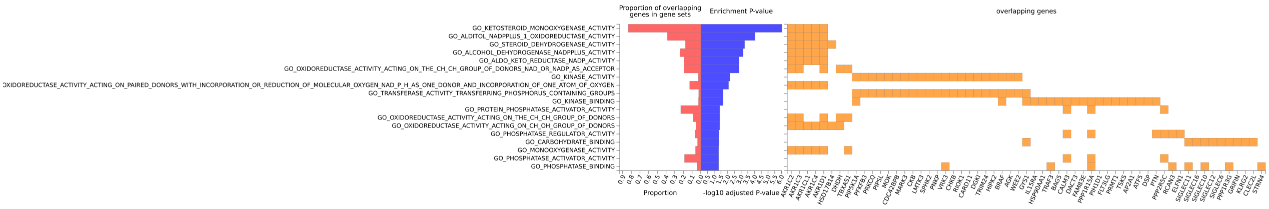

Figure 7: Functional enrichment of input genes in Gene Sets according to Molecular Function. This defines the molecular processes in which the enriched genes are involved.

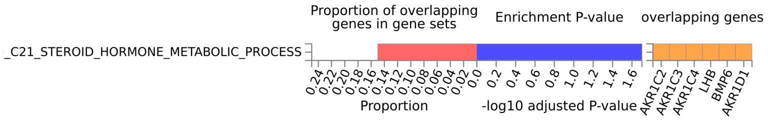

Figure 8: Functional enrichment of input genes in Gene Sets showing the biological processes in which the enriched genes are involved.

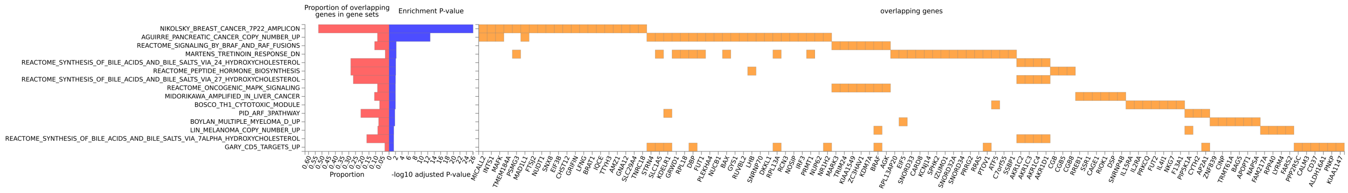

Figure 9: Functional enrichment of input genes using curated Gene Sets. These shows functions and pathways of the enriched genes based on curated gene sets from public databases

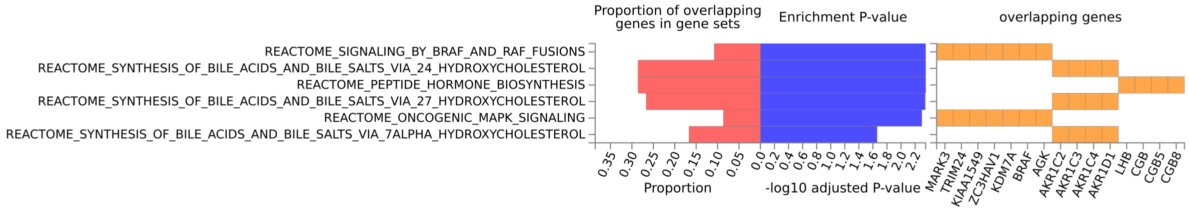

Figure 10: Functional enrichment of input genes in Gene Sets using the Reactome database. Functional consequence of the enriched genes were extracted from the reactome database.

4 Appendix C1: Assessing the predictive behavior of ensembles genetic prognostic models in predicting future worsening outcomes in MS over time

Table 1: Variable Selection, Training, and Validation of Ensemble Genetic Classifiers for the prediction of MS disability worsening over time

Selection Model	Prediction Model	Area under receiver operating characteristic curve (AUC) and Positive predictive value (PPV)			
		Visits: 1→4	Visits: 1→7	Visits: 1→10	Visits: 1→12
		DD≤5year N_{subj}=45; N_{obs}=215	DD≤10years N_{subj}=55; N_{obs}=285	DD≤15years N_{subj}=60; N_{obs}=375	DD≤20years N_{subj}=67 N_{obs}=415
**LASSO** (#SNPs = 141)	MEgbm	0.79(0.72, 0.87)	0.72(0.66, 0.77)	0.67(0.62, 0.72)	0.67(0.62, 0.72)
		0.92(0.85, 0.99)	0.86(0.81, 0.92)	0.79(0.74, 0.84)	0.78(0.73, 0.82)
	MErf	0.81(0.74, 0.88)	0.68(0.62, 0.74)	0.64(0.59, 0.70)	0.64(0.59, 0.69)
		0.88(0.81, 0.95)	0.85(0.79, 0.90)	0.80(0.76, 0.85)	0.79(0.75, 0.84)
	GBM	0.51(0.41, 0.60)	0.53(0.47, 0.60)	0.51(0.46, 0.57)	0.52(0.47, 0.58)
		0.64(0.56, 0.72)	0.67(0.61, 0.72)	0.60(0.55, 0.65)	0.57(0.52, 0.61)
	RF	0.50(0.41, 0.59)	0.53(0.46, 0.60)	0.52(0.46, 0.57)	0.52(0.47, 0.58)
		0.60(0.52, 0.68)	0.67(0.61, 0.72)	0.58(0.63, 0.63)	0.59(0.54, 0.63)
**EN** (#SNPs=150)	MEgbm	0.78(0.71, 0.86)	0.69(0.63, 0.75)	065(0.59, 0.70)	0.64(0.59, 0.69)
		0.90(0.83, 0.97)	0.84(0.79, 0.90)	0.79(0.75, 0.84)	0.78(0.73, 0.83)
	MErf	0.82(0.76, 0.89)	0.69(0.63, 0.74)	0.65(0.60, 0.70)	0.65(0.60, 0.70)
		0.92(0.85, 0.99)	0.56(0.80, 0.91)	0.84(0.79 0.88)	0.83(0.78, 0.89)
	GBM	0.53(0.43, 0.62)	0.54(0.47, 0.62)	0.52(0.45, 0.58)	0.53(0.48, 0.59)
		0.62(0.54, 0.70)	0.61(0.55, 0.66)	0.57(0.52, 0.62)	0.57(0.52, 0.62)
	RF	0.52(0.42, 0.61)	0.53(0.47, 0.60)	0.52(0.47, 0.58)	0.53(0.47, 0.59)
		0.61(0.53, 0.69)	0.60(0.55, 0.66)	0.58(0.53, 0.63)	0.58(0.53, 0.63)
**NNG-SIS** (#SNPs=130)	MEgbm	0.83(0.76, 0.89)	0.73(0.68, 0.79)	0.69(0.64, 0.74)	0.68(0.63, 0.73)
		0.91(0.84, 0.97)	0.83(0.78, 0.88)	0.79(0.75, 0.84)	0.78(0.73, 0.83)
	MErf	0.82(0.76, 0.89)	0.69(0.63, 0.75)	0.65(0.60, 0.70)	0.65(0.60, 0.70)
		0.93(0.86, 1.00)	0.92(0.86, 0.97)	0.86(0.82, 0.91)	0.87(0.82, 0.91)
	GBM	0.50(0.41, 0.60)	0.52(0.45, 0.58)	0.51(0.46, 0.57)	0.52(0.46, 0.57)
		0.60(0.51, 0.68)	0.61(0.56, 0.67)	0.57(0.52, 0.62)	0.58(0.53, 0.62)
	RF	0.53(0.43, 0.62)	0.50(0.44, 0.57)	0.50, 0.45, 0.56	0.50(0.45, 0.56)
		0.58(0.50, 0.66)	0.60(0.54, 0.65)	0.57(0.52, 0.61)	0.56(0.51, 0.61)

AUC: unshaded values.
PPV: shaded values.
DD: Disease duration
Values in brackets are the 95% C.I.
#SNPs: Total number of SNPs selected.

5 Appendix C2: Assessing the dynamic predictive behavior of the final ensemble with 28 prognostic variants

Table 2: Training and Validation of ensemble genetic models using 28 candidate risk variants. Time-dynamic AUC and 95% C.I are shown. MEgbm and MErf had the highest sensitivities, and are best suited for predicting worsening outcomes over time.

(a) GBM

Visits	Train AUC(C.I)	Test AUC(C.I)
1	0.59(0.49, 0.69)	0.56(0.48, 0.78)
2	0.57(0.47, 0.66)	0.61(0.46, 0.77)
3	0.54(0.45, 0.64)	0.73(0.58, 0.88)
4	0.52(0.42, 0.62)	0.60(0.44, 0.76)
5	0.52(0.42, 0.62)	0.50(0.33, 0.67)
6	0.56(0.45, 0.66)	0.50(0.28, 0.72)
7	0.61(0.50, 0.73)	0.57(0.33, 0.81)
8	0.51(0.38, 0.65)	0.59(0.38, 0.81)
9	0.51(0.36, 0.66)	0.60(0.40, 0.79)
10	0.61(0.42, 0.80)	0.78(0.54, 1.01)

(b) RF

Visits	Train AUC(C.I)	Test AUC(C.I)
1	0.89(0.83, 0.95)	0.66(0.41, 0.91)
2	0.86(0.80, 0.92)	0.55(0.39, 0.71)
3	0.88(0.83, 0.93)	0.67(0.52, 0.83)
4	0.88(0.82, 0.93)	0.54(0.37, 0.70)
5	0.82(0.75, 0.89)	0.56(0.39, 0.74)
6	0.88(0.81, 0.94)	0.60(0.40, 0.80)
7	0.71(0.61, 0.81)	0.62(0.38, 0.87)
8	0.60(0.46, 0.73)	0.54(0.31, 0.76)
9	0.64(0.49, 0.79)	0.52(0.31, 0.73)
10	0.68(0.48, 0.89)	0.63(0.37, 0.89)

(c) MEgbm

Visits	Train AUC(C.I)	Test AUC(C.I)
1	0.86(0.80, 0.92)	0.95(0.89, 1.02)
2	0.93(0.88, 0.97)	0.92(0.83, 1.00)
3	0.92(0.87, 0.96)	0.89(0.80, 0.97)
4	0.92(0.88, 0.97)	0.93(0.87, 1.00)
5	0.89(0.83, 0.94)	0.97(0.92, 1.02)
6	0.86(0.79, 0.92)	0.72(0.53, 0.90)
7	0.81(0.73, 0.90)	0.93(0.84, 1.02)
8	0.84(0.73, 0.94)	0.97(0.90, 1.03)
9	0.88(0.79, 0.96)	0.91(0.82, 1.01)
10	0.90(0.79, 1.01)	0.86(0.70, 1.03)

(d) MErf

Visits	Train AUC(C.I)	Test AUC(C.I)
1	0.99(0.98, 1.00)	0.65(0.37, 0.93)
2	0.99(0.98, 1.00)	0.87(0.78, 0.97)
3	0.99(0.98, 1.00)	0.63(0.47, 0.79)
4	0.98(0.97, 1.00)	0.72(0.57, 0.86)
5	0.98(0.95, 1.00)	0.69(0.54, 0.84)
6	0.97(0.94, 1.00)	0.55(0.32, 0.77)
7	0.92(0.85, 0.98)	0.82(0.66, 0.97)
8	0.88(0.80, 0.96)	0.68(0.48, 0.89)
9	0.92(0.85, 0.99)	0.65(0.46, 0.84)
10	0.93(0.85, 1.00)	0.67(0.41, 0.93)

6 Fixed effects estimates of the final ensemble prognostic model on the original dataset without EDSS imputation

Table 3: **Multivariable mixed-effects Cox model on original data (without imputation of EDSS) using 28 candidate genetic variants. These results are quite similar to those on Table 2 of the article.**

SNP	*CHR*	*POS*	*Alleles*	*P-value*	*HR*	*beta*	*SE*
$rs12722559^{\gamma_7}$	10	64449549	G/T	1.20E-04	0.94	-0.07	0.02
rs4808760	10	6070273	C/A	6.10E-04	0.91	-0.11	0.03
rs9277626	19	18301979	G/C	3.80E-02	0.92	-0.07	0.03
rs12434551	6	33081823	G/A	2.20E-03	0.93	-0.07	0.02
rs7260482	14	69253364	A/T	4.00E-02	1.06	0.07	0.03
rs12588969	19	45143942	A/C	1.30E-01	0.91	-0.1	0.06
rs6032662	14	103230758	C/G	2.10E-10	1.11	0.09	0.01
rs11256593	20	44734310	C/T	9.30E-02	1.10	0.1	0.06
rs802730	10	6117322	T/C	5.10E-57	1.13	0.12	0.01
rs962052	6	128280104	T/C	1.10E-02	1.06	0.06	0.02
rs1465697	2	151644203	C/T	9.70E-02	1.06	0.07	0.04
rs2590438	19	49837246	C/T	1.70E-128	1.09	0.09	0.00
rs1801133	3	187565968	T/G	3.60E-03	1.10	0.09	0.03
rs11852059	1	11856378	A/G	1.70E-02	0.94	-0.06	0.03
rs531612	14	52306091	A/C	8.80E-02	0.93	-0.07	0.04
rs12925972	11	65705432	C/T	3.00E-02	1.06	0.08	0.04
rs1177228	16	79111297	C/T	6.60E-04	1.08	0.09	0.03
rs2286974	2	61242410	G/A	4.10E-03	1.09	0.09	0.03
rs2705616	16	11114512	G/A	8.50E-02	1.11	0.11	0.06
rs58166386	4	87862396	G/C	2.30E-04	0.87	-0.11	0.03
rs10271373	19	16559421	G/A	1.50E-04	1.08	0.08	0.02
rs72989863	7	138729795	C/A	1.10E-07	0.91	-0.1	0.02
$rs55858457^{\gamma_5}$	4	164493807	G/A	4.10E-03	1.07	0.07	0.02
rs12211604	7	2443302	G/T	3.70E-07	0.93	-0.07	0.01
$rs6533052^{\gamma_3}$	6	7100029	A/G	3.20E-07	1.16	0.15	0.03
$rs7731626^{\gamma_2}$	4	103911781	A/G	4.40E-03	0.96	-0.05	0.02
$rs6589939^{\gamma_4}$	5	55444683	G/A	2.40E-05	0.93	-0.08	0.02
